# Neoantigen-Specific T-Cell Immune Responses: The Paradigm of *NPM1*-Mutated Acute Myeloid Leukemia

**DOI:** 10.3390/ijms22179159

**Published:** 2021-08-25

**Authors:** Fabio Forghieri, Giovanni Riva, Ivana Lagreca, Patrizia Barozzi, Francesca Bettelli, Ambra Paolini, Vincenzo Nasillo, Beatrice Lusenti, Valeria Pioli, Davide Giusti, Andrea Gilioli, Corrado Colasante, Laura Galassi, Hillary Catellani, Francesca Donatelli, Annalisa Talami, Rossana Maffei, Silvia Martinelli, Leonardo Potenza, Roberto Marasca, Enrico Tagliafico, Rossella Manfredini, Tommaso Trenti, Patrizia Comoli, Mario Luppi

**Affiliations:** 1 Section of Hematology, Department of Medical and Surgical Sciences, University of Modena and Reggio Emilia, Azienda Ospedaliero-Universitaria di Modena, Policlinico, 41124 Modena, Italy; ivana.lagreca@unimore.it (I.L.); patrizia.barozzi@unimore.it (P.B.); francesca.bettelli@unimore.it (F.B.); paolini.ambra@aou.mo.it (A.P.); pioli.valeria@aou.mo.it (V.P.); davide.giusti@unimore.it (D.G.); gilioli.andrea@aou.mo.it (A.G.); colasantecorrado@gmail.com (C.C.); l.galassi1591@gmail.com (L.G.); hillary.catellani@gmail.com (H.C.); donatelli.fran@gmail.com (F.D.); annalisa.talami@gmail.com (A.T.); rossana.maffei@unimore.it (R.M.); silvia.martinelli@unimore.it (S.M.); leonardo.potenza@unimore.it (L.P.); roberto.marasca@unimore.it (R.M.); 2 Department of Laboratory Medicine and Pathology, Unità Sanitaria Locale, 41126 Modena, Italy; g.riva@ausl.mo.it (G.R.); vincenzo.nasillo@unimore.it (V.N.); beatrice.lusenti@gmail.com (B.L.); t.trenti@ausl.mo.it (T.T.); 3 Center for Genome Research, Department of Medical and Surgical Sciences, University of Modena and Reggio Emilia, Azienda Ospedaliero-Universitaria di Modena, 41124 Modena, Italy; enrico.tagliafico@unimore.it; 4 Center for Regenerative Medicine “Stefano Ferrari”, Department of Life Sciences, University of Modena and Reggio Emilia, 41125 Modena, Italy; rossella.manfredini@unimore.it; 5 Pediatric Hematology/Oncology Unit and Cell Factory, Istituto di Ricovero e Cura a Carattere Scientifico (IRCCS) Policlinico San Matteo, 27100 Pavia, Italy; pcomoli@smatteo.pv.it

**Keywords:** *NPM1* mutation, acute myeloid leukemia, leukemia-specific neoantigen, NPM1-mutated-specific T cells, adoptive immunotherapy, immune-checkpoint inhibitors

## Abstract

The C-terminal aminoacidic sequence from NPM1-mutated protein, absent in normal human tissues, may serve as a leukemia-specific antigen and can be considered an ideal target for *NPM1*-mutated acute myeloid leukemia (AML) immunotherapy. Different in silico instruments and in vitro/ex vivo immunological platforms have identified the most immunogenic epitopes from NPM1-mutated protein. Spontaneous development of endogenous NPM1-mutated-specific cytotoxic T cells has been observed in patients, potentially contributing to remission maintenance and prolonged survival. Genetically engineered T cells, namely CAR-T or TCR-transduced T cells, directed against NPM1-mutated peptides bound to HLA could prospectively represent a promising therapeutic approach. Although either adoptive or vaccine-based immunotherapies are unlikely to be highly effective in patients with full-blown leukemia, these strategies, potentially in combination with immune-checkpoint inhibitors, could be promising in maintaining remission or preemptively eradicating persistent measurable residual disease, mainly in patients ineligible for allogeneic hematopoietic stem cell transplant (HSCT). Alternatively, neoantigen-specific donor lymphocyte infusion derived from healthy donors and targeting NPM1-mutated protein to selectively elicit graft-versus-leukemia effect may represent an attractive option in subjects experiencing post-HSCT relapse. Future studies are warranted to further investigate dynamics of NPM1-mutated-specific immunity and explore whether novel individualized immunotherapies may have potential clinical utility in *NPM1*-mutated AML patients.

## 1. Introduction

The graft-versus-leukemia (GvL) effect associated with allogeneic hematopoietic stem cell transplantation (HSCT) and the efficacy of donor lymphocyte infusion (DLI) to eradicate residual disease after HSCT still actually represent cornerstones of immunotherapy for the treatment of acute myeloid leukemia (AML) [1]. However, apart from mediating the beneficial GvL effect, the immune system, mainly through antigen-reactive T cells, may also induce graft-versus-host disease (GvHD), leading to potentially harmful post-transplant complications. These observations suggest the need for innovative suitable immunotherapeutic approaches aiming to obtain robust anti-leukemic activity while avoiding T-cell cytotoxicity directed against healthy tissues [1]. Of interest, harnessing antigen-specific anti-leukemic T-cell activity, minimizing the risk of “on-target/off-tumor” toxicity, should also increasingly be translated into AML management outside the allogeneic HSCT setting, but certainly still represents a clinical challenge [1,2]. Any ideal target antigen for AML immunotherapy should display strong and homogeneous expression levels in most to all leukemic cells, potentially including leukemic stem cell (LSC) subpopulation, with minimal to absent expression in normal hematopoietic cells and extramedullary tissues. Furthermore, optimal target antigens should be expressed in most AML cases, showing a clearly defined indispensable leukemogenic role and possibly harboring strong immunogenic properties. While leukemia-associated antigens (LAA) are overexpressed on AML cells relative to normal tissues but are not usually lineage-specific and may also be found on non-hematopoietic cells, leukemia-specific antigens, resulting from aberrant proteins encoded by ideally leukemogenic mutations, are exclusively expressed in malignant clones, therefore representing optimal candidate targets for anti-leukemic immunity [1,2,3]. Indeed, neoantigens are composed of peptides derived from full-length leukemia-specific proteins through a multistep intracellular process, eventually resulting in the antigen presentation on the cell surface in the context of Human Leukocyte Antigen (HLA) molecules, with the subsequent potential recognition of peptide-HLA complex by specific T-cell receptor (TCR) [3]. However, it should be noted that not all intracellular neoantigens derived from leukemia-specific gene lesions are finally presented on the cell surface and, in addition, that aberrant proteins will not necessarily yield target neoantigens [1,3]. Furthermore, some neoantigens could be encoded by patient-specific passenger mutations, which could be lost due to immune editing, a phenomenon especially observed in solid tumors with higher mutational load, resulting in tumor immune evasion, which could also be observed in case of either altered proteosomal processing of the immunogenic epitope or down-regulation/loss of HLA molecules expression [3,4]. Conversely, the neoantigens derived from driver gene mutations directly leukemogenic are less likely to induce immune evasion because leukemic cells need to definitely express the critical driver mutated protein in order to maintain their malignant phenotype [3,4]. Relevant to this, nucleophosmin (*NPM1*) gene mutations, observed in nearly 30% of adult AML patients, accounting for approximately 50–60% of cases among the cytogenetically normal AML subgroup, represent one of the most frequent genetic lesions documented in AML [5,6,7,8]. Moreover, *NPM1* mutations are highly specific, being almost exclusively found in AML, and generally expressed in the entire leukemic population, while not detectable in clonal hematopoiesis [5,7,8,9,10]. As expected for driver genetic lesions, *NPM1* mutations are also stable throughout the course of the disease, with most relapses being due to the recurrence of the original *NPM1*-mutated clone, whereas only 5% to 10% of recurrent cases are characterized by the absence of *NPM1* mutations. Of note, in these cases, the hypothesis of development of a second different AML favored by the persistence of clonal hematopoiesis following the eradication of the original *NPM1*-mutated clone is actually considered [7,8,11,12,13]. Most significantly, *NPM1* mutations result in structural modifications of the C-terminus of NPM1 protein, with consequent abnormal cytoplasmic delocalization, leading to alterations of multiple cellular pathways, critical for leukemic transformation [5,7,8,14]. NPM1 cytoplasmic dislocation could also favor protein processing and degradation pathways, presumptively determining more efficient HLA presentation [15]. Additionally, no aminoacidic sequences from normal human tissues present in databanks match that of the 11 residues from the C-terminal NPM1-mutated protein, suggesting that this aminoacidic sequence may clearly serve as a leukemia-specific antigen [15]. Based upon the above indicated biological requirements, NPM1-mutated protein may thus be considered an ideal target antigen for AML immunotherapy [1,2].

## 2. Identification of Most Immunogenic Peptides from NPM1-Mutated Protein

Liso et al., proposed for the first time that the unique C-terminal sequences of NPM1-mutated protein may represent a potential immunotherapeutic target [15]. Indeed, the in silico analysis by Epimatrix System predicted that several peptide sequences from NPM1-mutated protein could potentially be presented by common HLA class I and II molecules. Moreover, they investigated the capacity of candidate peptides to bind HLA molecules in vitro, showing that two of the selected peptides, namely CLAVEEVSL and AIQDLCLAV, both deriving from *NPM1* mutation types A and D, efficiently bound to HLA-A2 molecules, similarly to the reference control peptide obtained from the Epstein–Barr virus BMLF1 protein (Table 1) [15]. Greiner et al., subsequently screened the whole amino acid sequences of mutated (types A, B, C, D) and wild-type NPM1 protein to identify HLA-A*02:01-binding T-cell epitopes utilizing the SYFPEITHI, Rankpep, and HLA-Bind software programs [16]. The ten 9-mer peptides yielding the highest predictive scores for HLA-binding, retrieved by the three different bioinformatics algorithms, were therefore evaluated in an 8-day culture setting in which CD8+ T cells obtained from peripheral blood (PB) of healthy subjects and AML patients, were ex vivo stimulated with antigen-presenting cells (APCs) pulsed with each individual peptide, and tested for cytokine secretion capability. A significant increase of specific CD8+ T cells secreting Interferon-γ (IFNγ) and granzyme B in response to the most immunogenic HLA-A2-restricted NPM1-mutated peptides, #1 AIQDLCLAV, and #3 AIQDLCVAV, was observed by Enzyme-linked immunospot (ELISPOT) assay (Table 1) [16,17]. Although both peptides were naturally processed and recognized by specific CD8+ T cells, among 27 *NPM1*-mutated AML patients a significantly higher frequency of T-cell responses was shown against peptide #3 (44% of patients) compared to healthy subjects (6/33, 18%), whereas for peptide #1 the frequency of specific immune responses found in *NPM1*-mutated AML and healthy volunteers (33% and 39%, respectively) was not statistically different [16]. Additional immunoepitopes derived from NPM1-mutated protein and restricted to different HLA class I molecules were predicted by Kuzelova et al., using the Immune Epitope Database (IEDB), as detailed in Table 1 [18]. The generation of high-affinity binding immunoepitopes involving several amino acid sequences from the unmutated portion of NPM1 protein was also identified by the authors [18]. On the other hand, Forghieri et al., later documented the emergence of NPM1-mutated-specific IFNγ-secreting T cells in 34 of 52 (65.4%) PB samples obtained from 17 adults with *NPM1*-mutated AML, by ELISPOT assay after 20 hour antigenic stimulation with a comprehensive mixture of 18 (9–18 mers) peptides spanning the C-terminal of NPM1-mutated protein [19]. Subsequently, in order to ex vivo identify the most immunogenic epitopes, peptide mixtures were progressively split, until the ELISPOT assay was performed using single individual peptides, as antigenic stimulation, on the same samples stored from 12 of the 17 patients, which were previously stimulated with the entire 18-peptide mixture. These extensive examinations permitted us to identify LAVEEVSLR (13.9) and AVEEVSLRK (14.9) as the most immunogenic 9-mer peptides within the C-terminal of NPM1-mutated protein (Table 1). Accordingly, ELISPOT assay, after ex vivo stimulation with the combination of 13.9 and 14.9 peptides, revealed NPM1-mutated-specific T cells secreting IFNγ in 43/85 (50.6%) PB samples and in 34/80 (42.5%) bone marrow (BM) samples, collected from 26 *NPM1*-mutated AML patients. No significant differences in either frequency of positive samples or magnitude of specific T-cell responses were observed after stimulation when PB and BM samples were compared. Overall, the spontaneous occurrence of NPM1-mutated-specific immune responses was shown in 26 of 31 (83.9%) patients from our series [19]. Of interest, after stimulation with the combination of 13.9 and 14.9 peptides, IFNγ-secreting NPM1-mutated-specific T cells could also be documented by ELISPOT assay in PB samples of 3 out of 11 (27.3%) healthy individuals. The 14.9 peptide, recognized to have in silico binding affinity at least for HLA-A*02:01, A*03:01, A*11:01, and A*68:01, may show significant advantages based on its extreme C-terminal aminoacidic sequence, which is shared by most frequent *NPM1* mutation types, namely A/D, B, and C (Table 1). These features could thus facilitate documentation of specific immune responses, irrespective of *NPM1* mutation type [15,16,18,19]. Our ELISPOT assay, performed after brief ex vivo antigenic stimulation, markedly differed from the ELISPOT analysis carried out after 8-day culture by Greiner et al., and this feature may account for diverse aminoacid sequences being identified as the most immunogenic [16,19]. Subsequently, van der Lee et al., immunoprecipitated peptide/HLA class I surface molecules from 12 primary AML samples, eluted the peptides from the binding groove, and analyzed the peptidome by tandem mass spectrometry [20]. Among the five different peptides identified from the alternative reading frame of NPM1-mutated protein by searching the HLA class I ligandome, as detailed in Table 1, CLAVEEVSL peptide, predicted to bind to the frequently expressed in the Caucasian population HLA-A*02:01, was selected for further immunological investigations [20]. Specifically, PB samples from six HLA-A*02:01-positive *NPM1*-mutated AML patients were analyzed for immune responses against CLAVEEVSL, but tetramer-positive T cells were not detected, possibly indicating that frequencies of specific immune response naturally occurring in vivo in these patients having achieved complete remission (CR) after chemotherapy, could be below the threshold of detection. Conversely, NPM1-mutated tetramer-positive CD8+ T cells were successfully isolated from PB samples of six HLA-A*02:01-positive healthy individuals, with subsequent expansion of 13 T-cell clones positive for the NPM1-mutated CLAVEEVSL tetramer. Two of these latter clones showed specific reactivity, with IFNγ release, against HLA-A*02:01-positive T2 cells exogenously pulsed with CLAVEEVSL peptide, whereas no reactivity was documented against T2 cells when pulsed with an irrelevant HLA-A*02:01-binding CMV control peptide. Moreover, a minority of T-cell clones yielded ex vivo reaction against HLA-A*02:01-positive primary blasts from AML patients harboring *NPM1* gene mutations, whereas no T-cell reactivity was observed against HLA-A*02:01-positive AML with wild-type *NPM1* [20]. Furthermore, Narayan et al. utilized NetMHC to investigate HLA class I binding affinities for putative 9–11 mer peptides spanning common recurrent AML mutations and computationally documented that several NPM1-mutated peptides, including mutation-bearing sequences AIQDLCLAV and AVEEVSLRK, are predicted to efficiently bind various HLA class I alleles [21]. Additionally, the authors empirically measured, by mass spectrometry, the HLA class I and class II immunopeptidomes of 13 primary leukemic samples and two AML cell lines, namely OCI-AML3 and MV4-11, carrying type A *NPM1* mutation and *FLT3*-ITD, respectively. Two endogenous mutation-bearing HLA class I 9-mer ligands from NPM1-mutated sequences, namely AVEEVSLRK from two patient samples and C(Cys)LAVEEVSL from OCI-AML3 cell line were predicted to bind common HLA haplotypes, as detailed in Table 1. Of interest, as previously reported by Kuzelova et al. [18], non-mutation-bearing ligands from NPM1 protein were also frequently found in either patient AML samples or cell lines, including ligands close to or corresponding to hotspot mutation regions. Whether the processing and presentation of non-mutation-bearing HLA ligands from wild-type regions of the protein may subsequently increase the likelihood of NPM1-mutated peptides to be processed and presented still need to be elucidated [21]. Moreover, it was previously described that recipients of allogeneic HSCT who develop extensive GvHD are able to generate immune responses against wild-type NPM1 protein [22], while cytotoxic T-lymphocyte (CTL) lines derived from colorectal cancer patients may also recognize normal NPM1 protein sequences [23]. These observations collectively support the general immunogenicity of both mutated and non-mutated peptide sequences from NPM1 protein [15,16,18,19,20,21,22,23].

## 3. Cytolytic Activity of NPM1-Mutated-Specific T Cells in Ex Vivo Assays

To assess peptide recognition and antigen-specific cell lysis, Greiner et al., generated NPM1-mutated-specific CTLs from the PB of four healthy subjects [16]. CD8+ T cells were isolated and stimulated weekly with NPM1-mutated peptide #1. Antigen-specific cytotoxic activity was documented by Cr^51^-release assays on day 21, using exogenously peptide-pulsed T2 cells as target cells. Furthermore, the cytolytic potential of NPM1-mutated-specific CTLs was demonstrated against primary *NPM1*-mutated leukemic blasts, in the context of HLA-A2, whereas blasts from patients without *NPM1* mutations or lacking HLA-A2 expression were not recognized. These data showed that NPM1-mutated-derived epitope peptides can be naturally processed by AML blasts and efficiently recognized, at least in the context of HLA-A2 [16]. Forghieri et al., interestingly performed phenotypic and functional characterization, by Cytokine Secretion Assays (CSA), of NPM1-mutated-specific T cells, identifying a subset of degranulation marker CD107a-positive cytotoxic IFNγ-producing T cells among both CD8+ and CD4+ populations from PB and BM [19]. The analysis of memory T-cell profiles also showed that Central Memory (CM) and Effector Memory (EM) T-cell phenotypes were equally distributed among Tumor Necrosis Factor α (TNFα)-producing T cells, whereas EM T and CM phenotypes, both CD4+ and CD8+, were predominantly identified among IFNγ-producing T cells and IL2-producing T cells, respectively [19]. Moreover, by stimulation with dendritic cells pulsed with different NPM1-mutated peptide mixtures in a 13-day culture, we were able to expand ex vivo leukemia-specific CD8+ and CD4+ CTLs from four *NPM1*-mutated AML patients, as well as to prime leukemia-specific responses in three healthy donors. Among the different NPM1-mutated-peptide pools employed, the combination of 13.9 and 14.9 peptides with the addition of the 11-mer CLAVEEVSLRK peptide was mostly able to elicit specific lytic activity against targets represented by autologous PHA blasts pulsed with NPM1-mutated-derived peptides, in all the subjects tested, which was comparable to cytotoxicity observed by using a comprehensive mixture containing all 18 NPM1-mutated peptides. The ability of NPM1-mutated-specific CTLs, stimulated and expanded from either patients or healthy volunteers, to recognize and exert direct lytic activity against either autologous or allogeneic primary leukemic blasts, respectively, was also demonstrated [19]. Of interest, the presence of the 11-mer peptide along with 13.9 and 14.9 epitopes allowed the stimulation of leukemia-specific CD4+ T cells, which have previously been shown to directly induce potent anti-tumor, HLA class II-mediated, cytotoxic responses in vivo, in addition to providing help to CD8+ CTLs [19,26].

## 4. Clinical Significance of NPM1-Mutated-Specific Immune Responses in AML Patients

In a survival analysis of 25 patients affected with *NPM1*-mutated AML, Greiner et al., documented a better overall survival (OS) in patients experiencing autologous specific T-cell responses against one or two immunogenic NPM1-mutated epitopes, namely peptides #1 and #3, compared to cases showing no specific immune responses, suggesting that immunity against the mutated region of NPM1 protein may potentially contribute to the globally favorable outcome of *NPM1*-mutated AML patients [27]. To further confirm the hypothesis that efficient responses against immunogenic NPM1-mutated epitopes may be induced in humans, Kuzelova et al., identified a skewed HLA molecule distribution in AML patients and documented that subjects expressing HLA alleles suitable for actively presenting NPM1-derived peptides are less prone to develop *NPM1*-mutated AML [18]. Indeed, a few HLA class I alleles, mainly B*07, B*18, and B*40, exhibited a surprisingly reduced incidence in the *NPM1*-mutated patient group compared to controls, namely healthy subjects, and *NPM1*-wild-type AML patients. Therefore, it has been indirectly suggested that specific immune responses to the NPM1 protein could protect from AML occurrence in a large part of individuals who express appropriate HLA alleles and may help in maintaining sustained and durable responses in the remaining cases, who unfortunately develop AML, despite bearing at least one of those depleted alleles [18]. Subsequently, in a larger cohort of 398 patients, the same authors found that HLA-A*02, B*07, B*40, and C*07:01 alleles were under-represented in *NPM1*-mutated AML compared to the healthy population, further supporting the epidemiological hypothesis that anti-NPM1 protein immune reaction could contain AML development. Candidate immunoepitope peptides derived from either mutated NPM1 sequences for HLA-A*02:01 (Table 1) or unmutated NPM1 protein for HLA B*40 and B*07 were observed using prediction software tools. Moreover, the presence of B*07 or C*07:01 antigen only was associated with better survival outcomes in *NPM1*-mutated AML patients without *FLT3*-ITD [25]. Subsequently, in order to evaluate the dynamics of specific immune responses throughout the disease course, Forghieri et al., collected PB and BM samples from *NPM1*-mutated AML patients at different timepoints [19], whereas in the former study by Greiner et al., the presence of NPM1-mutated-specific T cells was investigated in a single PB sample per patient [16]. In our cohort, increased and sustained specific immune responses were commonly observed in patients with long-term CR, especially in cases experiencing persistent molecular CR, whereas either decreased number or absence of IFNγ-producing NPM1-mutated-specific T cells strongly correlated with molecular or morphologic leukemia relapse [19], suggesting an inverse correlation between the kinetics of measurable residual disease (MRD) monitored by real-time quantitative polymerase chain reaction (RQ-PCR) for *NPM1*-mutated transcripts [28,29,30,31,32,33,34] and anti-leukemic specific T cells, as previously observed in Philadelphia chromosome-positive B-acute lymphoblastic leukemia patients [35,36,37]. Of interest, some cases from our series exhibited NPM1-mutated-reactive T-cell responses five years or later after the completion of anti-leukemic treatments, further suggesting that specific immune responses could have a central role in the long-term favorable clinical outcomes, at least in some *NPM1*-mutated AML patients [19]. Since *NPM1* mutations are present in the whole leukemic population, including LSCs from *NPM1*-mutated AML patients, it may be hypothesized that immune responses against NPM1-mutated protein could contribute to definitive eradication of MRD [7,19,38,39]. Moreover, robust NPM1-mutated specific T-cell responses were identified early in most patients, whose samples were available after having achieved morphologic CR by remission induction treatments including anthracycline, a prototype of immunogenic chemotherapy [19,40,41,42]. This latter observation is not surprising since it has recently been reported that infiltration by T-cell population, using immunohistochemical examinations, appeared to be quantitatively preserved in BM specimens from AML patients compared with healthy donors, even though an increased proportion of T-regulatory cells (Tregs) and higher frequency of PD1/CD8+ T cells coexpressing TIM3 or LAG3 immune-checkpoint molecules were documented by flow cytometry in AML patients, mainly in relapsed/refractory cases [43,44]. Moreover, in the study by Knaus et al., CD8+ T cells at AML diagnosis exhibited features of exhaustion and senescence, with AML blasts directly altering viability and expression of co-signaling molecules on CD8+ T cells. Following anti-leukemic treatments, phenotypic and transcriptional profiles of dysfunctional CD8+ T cells diverged between responders and non-responders. In particular, response to chemotherapy correlated with up-regulation of costimulatory and down-regulation of apoptotic and inhibitory T-cell signaling pathways, indicating plasticity and restoration of T-cell function. These observations collectively characterized the potential reversibility of T-cell dysfunction in AML patients who obtain response after intensive therapeutic approaches [43,44,45]. Interestingly, Alsuliman et al., also studied, in AML patients, long-lived viral-specific drug-effluxing CD4+ T cells, characterized as CD161+ CD95+ CD45RA- CD127hi CD28+ CD25int, with a distinct chemokine profile and a Th1-polarized proinflammatory phenotype [46]. The identification of this quiescent pathogen-specific CD4+ T-cell subpopulation, which was resistant to chemotherapy-induced cytotoxicity and subsequently expanded in AML patients, formerly rendered lymphopenic by chemotherapy, therefore contributing to repopulation and maintenance of anti-viral immunity, may spur further investigations on similar mechanisms that could preserve and sustain the emergence of endogenous long-lasting anti-leukemic specific immunity [46,47].

## 5. NPM1-Mutated-Specific T-Cell Responses in Allogeneic HSCT Setting

Forghieri et al., found a significantly higher magnitude of IFNγ-producing NPM1-mutated-specific T-cells in PB samples obtained in the allogeneic HSCT setting, compared to those observed in *NPM1*-mutated AML patients having received different consolidation strategies based on either autologous HSCT or chemotherapy only [19]. This interesting observation was not entirely surprising, since 27.3% of healthy donors from our cohort yielded IFNγ-producing NPM1-mutated-specific T cells by ELISPOT assay, and, in the study by Greiner et al., specific responses directed to peptides #1 and #3 were documented in 39% and 18% of healthy volunteers, respectively [16,19]. It should be acknowledged that these results from in vitro assays may possibly reflect the general capacity of T cells to be stimulated by foreign antigens [18], but we also intriguingly found that short sequences of four amino acids from the C-terminal of NPM1-mutated protein, namely LCLA, CLAV, LAVE, SLRK, and VEEV, are homologous with several common viral and bacterial antigens, potentially suggesting cross-reactive immune response mechanisms, as previously shown in the setting of malignant melanoma [19,48,49,50]. Moreover, van der Lee et al., documented immune responses and isolated CLAVEEVSL-specific TCR from HLA-A*02:01-positive healthy individuals, suggesting that high-affinity T cells against neoantigens arising from tumor-specific molecular lesions, such as *NPM1* mutations, which are absent in healthy tissues, are therefore not deleted by thymic selection through T-cell maturation processes [20,51,52]. These observations could collectively have relevant implications for *NPM1*-mutated AML patients undergoing allogeneic HSCT procedures. While allogeneic HSCT is generally recognized as the best therapeutic option in *NPM1*-mutated AML patients showing *FLT3*-ITD, at least in cases with a high allelic ratio; conversely, this procedure in first CR is generally not recommended in patients harboring *NPM1* gene mutations without *FLT3*-ITD [8,53,54]. However, allogeneic HSCT has been advocated by Rollig et al., as a potential therapeutic option for patients younger than 50 years, with low predicted transplant-related mortality and an HLA-identical donor, even though HSCT resulted in better relapse-free survival (RFS) only, with no significant improvement in OS, likely because patients could be salvaged by allogeneic HSCT in second CR [55]. However, either a suboptimal reduction in *NPM1*-mutated transcripts evaluated by RQ-PCR at relevant timepoints after chemotherapy or the occurrence of molecular relapse could potentially help in identifying patients with otherwise favorable genotype who may benefit from allogeneic HSCT [8,28,29,30,31,32,33,34]. Furthermore, recent studies interestingly demonstrated low relapse rates and more favorable outcomes in older *NPM1*-mutated and *FLT3*-ITD negative AML patients consolidated with allogeneic HSCT, challenging the paradigm of a chemotherapy-based consolidation being sufficient in older patient group [56,57]. Therefore, allogeneic HSCT in first CR should be at least considered and discussed in elderly fit AML patients with *NPM1*-mutated AML without *FLT3*-ITD to maximize their chances of cure [56,57]. Relevant to the allogeneic HSCT context, in a patient affected with *NPM1*-mutated AML in molecular relapse after allogeneic HSCT, described by a German group, preemptive DLI induced polyspecific CD8+ T cells responses directed to different LAA, including #1 and #3 NPM1-mutated peptides, which contributed to achievement of MRD negativity, thereby suggesting a correlation between GvL and LAA-specific CTL response [58]. Hofmann et al., subsequently assessed frequency and diversity of LAA-specific cytotoxic T cells in a cohort of 11 patients who had received allogeneic HSCT for different hematologic malignancies, including two *NPM1*-mutated AML cases in molecular relapse, before and after having received unmanipulated DLI [59]. For *NPM1*-mutated AML patients, NPM1-mutated epitopes, PRAME, RHAMM, proteinase 3, survivin 2, and WT1 were chosen as LAAs to be investigated. From the entire cohort, a significant increase in the number of LAAs actively recognized by CTLs and an enhanced LAA diversity in T-cell responses were detected in clinical responders following DLI when compared to non–responders. Moreover, clinical responders showed a significant reduction in the frequency of the highly immunosuppressive CD4+ Tregs. Of interest, one of the two *NPM1*-mutated AML patients developed NPM1-mutated-specific CTL response after administration of preemptive DLI and achieved molecular CR, whereas in the remaining clinically non–responder case, NPM1-mutated-specific CTLs were already detectable before DLI and persisted, but without showing an increase, after DLI [59]. Collectively, the authors suggested that increased specific T-cell responses against several different LAA, enhancing GvL effect and potentially able to target LSC population, as well as decreased numbers of Tregs after prophylactic/therapeutic DLI, could contribute to favorable clinical outcomes [59,60,61]. To maximize the GvL effect minimizing the risk of GvHD, Lulla et al., selectively activated and expanded stem cell donor-derived T cells reactive to multiple antigens expressed by AML/MDS cells, namely PRAME, WT1, survivin and NY-SEO-1 [62]. In contrast to DLI, leukemia-specific T cells selectively recognized and killed leukemia antigen-pulsed cells, with no activity against recipient’s normal cells in vitro. Additionally, anti-leukemic effects in vivo were demonstrated, with long-term post-HSCT remissions in the adjuvant patient group at a high risk of relapse (median leukemia-free survival not reached at a median follow-up of 1.9 years and estimated 2-year OS 77%), and objective responses observed in two out of eight patients with HSCT-refractory active disease. Therefore, allogeneic leukemia-specific T cells could represent a safe and promising preventative/therapeutic tool in the management of AML post-HSCT [62].

## 6. Exploiting Genetic Engineering of T Cells against *NPM1*-Mutated AML Cells

In recent years, different treatment platforms have overall been developed to harness anti-neoplastic T-cell activity in individuals affected with cancer, including hematologic malignancies: (a) recruitment of T cells independently of TCR specificity through T-cell-engaging antibody constructs, (b) reactivation of endogenous T-cell immune responses through either immune-checkpoint inhibitors (ICPIs) or other immunological strategies, and (c) genetic engineering of T cells, namely TCR-modified and chimeric antigen receptor (CAR) T cells, to be utilized as adoptive immunotherapy [1,63,64,65]. Relevant to this latter point, van der Lee et al., isolated and sequenced the CLAVEEVSL-specific TCR from one clone that specifically and strongly recognized HLA-A*02:01 peptide-pulsed targets and *NPM1*-mutated AML blasts [20]. Using a retroviral vector, this NPM1-mutated peptide-specific TCR was transferred into CD4+ and CD8+ T cells isolated from HLA-A*02:01-positive healthy individuals. T cells transduced with the transgenic NPM1-mutated neoantigen TCR were functional, resulting in specific recognition and release of IFNγ upon incubation with HLA-A*02:01-positive T2 cells loaded with CLAVEEVSL peptide, as well as with OCI-AML3 cell line and HLA-A*02:01-positive primary AML blasts harboring *NPM1* mutation. Moreover, CD8+, as well as CD4+ T cells transduced with the TCR for NPM1-mutated peptide, demonstrated efficient specific lysis by Cr^51^-release assay of *NPM1*-mutated, but not *NPM1* wild-type, HLA-A2-restricted primary leukemic blasts, indicating that CLAVEEVSL is a neoantigen that can be efficiently targeted on AML cells by NPM1-mutated sequence TCR gene transfer in a CD8 coreceptor-independent fashion. T cells transduced with TCR for NPM1-mutated protein also efficiently killed AML cells in an in vivo xenograft murine model, resulting in prolonged OS of immunodeficient NSG mice engrafted with HLA-A*02:01-positive *NPM1*-mutated OCI-AML3 human cells, in comparison to untreated mice or mice treated with CMV-specific TCR [20]. An advantage of gene therapy is the possibility to introduce TCR into distinct T-cell subsets with superior in vivo persistence, higher binding affinity to tumor antigens, and anti-leukemic efficacy. Conversely, gene therapy could potentially lead to immune escape of the tumor by HLA class I down-regulation or loss of antigen expression, even though this latter may not be a limitation clearly relevant to *NPM1* mutations, which are driver lesions essential for leukemogenesis and stably present in leukemic cells throughout the course of the disease [20]. Another specific drawback of TCR gene therapy is the risk of TCR chain mispairing between introduced and endogenous TCR α and β chain, resulting in reduced efficacy and potential toxicity by new TCRs with unknown specificities [4,20]. Intriguingly, Xie et al., recently used yeast surface display to isolate a single-chain variable fragment (scFv) specific for the NPM1-mutated neoepitope AIQDLCLAV in complex with HLA-A*02:01 (AIQ-HLA-A2) and subsequently developed CAR-T cells recognizing with high specificity and affinity AIQ-HLA-A2 complex, but not isolated HLA-A2 or HLA-A2 loaded with control peptides [4]. CAR-T cells exhibited potent in vitro cytotoxicity against HLA-A2-positive cell lines and primary blast cells expressing NPM1-mutated epitopes, but not against HLA-A2-positive leukemic cells without NPM1-mutated expression and different tumor cells in the absence of HLA-A2 expression, supporting the specificity of NPM1-mutated CAR-T cell recognition and the killing of target cells with AIQ-HLA-A2 complex on the cell surface. Of further interest, NSG mice injected intravenously with OCI-AML3 leukemic cells and receiving treatment with NPM1-mutated CAR-T cells showed a significant reduction in leukemia burden, resulting in prolonged survival compared to mice treated with untransduced T cells. NPM1-mutated CAR-T cells were thus capable of killing in vivo AML cells harboring HLA-A2-positivity and *NPM1* mutations, but not HLA-A2-positive lymphoma cells without NPM1-mutated protein, demonstrating killing specificity. CAR-T cells targeting the NPM1-mutated neoepitope were also effective in reducing primary HLA-A2-positive *NPM1*-mutated AML blast levels in patient-derived xenograft murine model [4]. NPM1-mutated CAR-T cells, as well as TCR transduced T cells, should be able to specifically target all leukemic cells without reacting against healthy tissues, including CD34+ hematopoietic stem/progenitor normal cells, due to the absence of NPM1-mutated aminoacidic sequence expression, hopefully yielding potent and highly specific anti-leukemic effect, minimizing tumor resistance and “on-target/off-tumor” toxicity [4,20]. In addition to either genetically engineered T cells against NPM1-mutated protein [4,20,24] or T cells reactive against patients’ primary blasts [66,67], the observation of spontaneous development of specific anti-leukemic T cell immunity directed against highly immunogenic NPM1-mutated peptides could also indicate the feasibility of stimulating and expanding ex vivo NPM1-mutated specific CTL lines from either patients with *NPM1*-mutated AML or healthy donors, who may be antigen-naive, to be potentially used for adoptive immunotherapeutic approaches [16,19,42].

## 7. Immune-Checkpoint Inhibitors and Novel Therapeutic Approaches in *NPM1*-Mutated AML

ICPIs recently changed clinical treatment algorithms of several solid tumors, especially melanoma and lung cancer, whereas the role of ICPIs has so far been less explored in hematologic malignancies, although anti-PD1 antibodies nivolumab and pembrolizumab are notable exceptions for the treatment of Hodgkin lymphoma and primary mediastinal B cell lymphoma [1]. Since in AML patients T-cell populations are globally preserved in BM, with increased frequencies of immune inhibitory and activating co-receptor expression, a potential role of T cell-harnessing therapies could be hypothesized in AML management [1,43,44,65]. However, single-agent ICPIs have so far demonstrated very modest anti-leukemic activity in clinical trials for relapsed/refractory AML patients, maybe due to suboptimal patient selection and lower mutational load of AML compared to solid tumors [1,68]. The role of several combination approaches of ICPIs with either hypomethylating agents (HMAs) or more intensive cytotoxic chemotherapy has recently been evaluated and is still under investigation in clinical trials enrolling AML patients at different disease stages, as comprehensively reviewed elsewhere [1,63,64,65,69]. Interestingly, Greiner et al., recently performed flow-cytometry and microarray analyses on a total of 30 AML samples, including 15 cases with *NPM1* mutations, to assess PD-L1 expression in leukemic cells at diagnosis [17,70]. Bulk leukemic cells of *NPM1*-mutated AML showed a significantly higher PD-L1 expression in comparison to *NPM1* wild-type cases. Remarkably, PD-L1 expression was detected at a higher percentage in leukemic progenitors/stem cell compartment (CD34+ CD38−) from *NPM1*-mutated AML cases than in patients without *NPM1* mutations [17,70]. In the retrospective series by Brodska et al., high PD-L1 expression in AML blasts predicted inferior survival outcomes, though this negative prognostic impact was limited to the patient subgroup affected with *NPM1*-mutated AML showing concurrent *FLT3*-ITD [71]. Intriguingly, Qin et al., discovered that normal NPM1 protein specifically binds to PD-L1 promoter in triple-negative breast cancer cells and activates PD-L1 transcription, therefore inhibiting T-cell activity in vitro and in vivo. Furthermore, they demonstrated that PARP1 suppresses PD-L1 transcription through its interaction with the nucleic acid-binding domain of NPM1, which is required for the binding of NPM1 at the PD-L1 promoter. Consistently, the PARP1 inhibitor olaparib elevated PD-L1 expression, arguing for potential combinatorial therapeutic strategies to enhance the efficacy of ICPIs, at least in this breast cancer subtype [72]. Furthermore, Greiner et al., investigated the influence of nivolumab and anti-CTLA4 antibody ipilumumab on specific immune response to several LAA, namely PRAME, RHAMM, WT1, and #3 peptide from NPM1-mutated protein, by specific T cells, stimulated from 12 AML patients, including five cases harboring *NPM1* mutations, against leukemic myeloid blasts and colony-forming cells including leukemic progenitor cells (CFC/LPC) [73]. In functional immunoassays using AML cell lines or primary HLA-A2-positive patient samples, the authors detected specific LAA-directed immune responses against CFC/LPC, which were significantly increased by the addition of nivolumab to CTL cultures, whereas no effect was observed when ipilimumab was added. Additionally, the combination of nivolumab and ipilimumab did not improve the inhibitory effect in cell colony growth compared to nivolumab alone. The anti-PD1-stimulated cytotoxic responses correlated to PD-L1 expression on leukemic cells, especially on progenitor cells [73]. The same authors, in a larger cohort of 15 *NPM1*-mutated and 15 *NPM1* wild-type AML patients, analyzed the influence of anti-PD1 on antigen-specific immune responses exerted by allogeneic CTLs against CFC/LPC in functional T-cell assays and colony-forming immunoassays [74,75]. A reduction in cell colonies in colony-forming immunoassays was demonstrated by CTLs against PRAME, RHAMM, and WT1 antigens in both *NPM1*-mutated and wild-type subgroups, whereas no response was exerted by CTLs against NPM1-mutated epitope in *NPM1* wild-type AML cases. Remarkably, the immune effect on CFC/LPC in *NPM1*-mutated AML patients markedly increased with specific CTLs against NPM1-mutated epitope and was stronger with the addition of anti-PD1 antibody to colony-forming immunoassays. Of note, anti-PD1 may overcome the immune resistance since a favorable effect was observed even in those cases not showing any colony-forming reduction in the presence of NPM1-mutated-specific T cells alone [74,75]. These data collectively confirmed the immunogenicity of neoantigens derived from NPM1-mutated protein, with the possible combination of immune-checkpoint inhibition targeting PD1/PD-L1 axis to enhance NPM1-mutated-specific immune responses in antigen-directed immunotherapeutic approaches [70,74,75]. While older unfit patients affected with *NPM1*-mutated AML rarely show long-term response to single HMAs, the combination of BCL-2 inhibitor venetoclax with HMAs, preferably 5-azacitidine, is currently emerging as the standard of care in this clinical setting, inducing CR in 70–90% of cases and significant survival advantages compared to alternative therapeutic strategies [7,8,76,77,78]. Perhaps, also in fit elderly patients with *NPM1*-mutated AML, venetoclax-based regimens could have the potential to challenge, as frontline treatment, standard intensive chemotherapy, which results in only 15% to 20% long-term survival [7,8,78]. Relevant to potential immunological implications, it is widely recognized that HMAs globally possess both immunostimulatory and immunosuppressive properties. In particular, they could stimulate immune responses against AML blasts by increasing the expression of cancer testis antigens, as well as relevant elements of the antigen-presenting machinery, such as HLA class I molecules and costimulatory molecules. On the other hand, HMAs can lead to immune escape of AML blasts through up-regulation of immune checkpoints and their ligands, as well as regulatory T cells. Combining the immunomodulatory effects of HMAs with other forms of immunotherapy may hold the promise of a synergistic effect on the immune system, leading to immunogenic antigen recognition and elimination of AML blasts [79]. More interestingly, Lee et al., recently discovered that venetoclax directly enhanced the effector function of double-negative T cells, used as surrogate of leukemia-specific T cells, and CD8+ T cells from healthy donors by increasing reactive-oxygen species generation, thereby increasing cytotoxicity against AML cells in vitro and in vivo [80]. In addition, 5-azacitidine rendered leukemic cells more susceptible to T-cell-mediated cytotoxicity via induction of viral mimicry. These results may collectively support the importance of T-cell immunity in facilitating the anti-leukemic effect induced by the combination therapy of venetoclax and 5-azacitidine [80]. It may be speculated that the relatively favorable outcomes observed in patients suffering from the highly immunogenic *NPM1*-mutated AML and receiving this frontline therapeutic regimen could be, at least in part, the result of immune-mediated mechanisms. Whether anti-leukemic NPM1-mutated-specific T-cell responses may be elicited in patients by the combination of venetoclax and HMAs needs to be further elucidated in prospective clinical studies. Besides the emergence of NPM1-mutated-specific T cells responses, other different immunotherapeutic approaches targeting antigens expressed on leukemic cells should be considered in *NPM1*-mutated AML. Given the association between high CD33 expression levels and *NPM1* mutations, the addition of gemtuzumab ozogamicin (GO) to standard chemotherapy has formerly been considered of potential benefit in this AML molecular subgroup [81]. A meta-analysis clearly demonstrated a survival benefit of GO added to standard chemotherapy for patients with AML patients either favorable or intermediate-risk cytogenetics [82]. In the ALFA-0701 clinical trial, the subset analyses pointed out the benefit of the addition of GO on 2-year event-free survival (EFS), RFS and OS in *NPM1*-mutated AML patients, mainly in the subgroup presenting with activating signaling mutations [83], while the AMLSG 09-09 German phase III study showed an improved RFS and a reduced cumulative incidence of relapse in *NPM1*-mutated AML, due to deeper reduction of *NPM1-*mutated transcript levels across all treatment cycles [84,85]. However, EFS, the early primary end-point of this trial, was not met due to excessive toxicity, possibly related to monoclonal antibody dosing strategy and inclusion in the regimens of *all-trans* retinoic acid (ATRA) and etoposide. GO benefit in this latter clinical trial was mostly observed in ≤70 year-old females without *FLT3*-ITD [84,85]. Globally, these findings support the possibility to incorporate in clinical practice GO into the frontline treatment of *NPM1*-mutated AML [82,83,84,85,86]. CD123, the alpha-subunit of interleukin-3 receptor, may represent another attractive target for immunotherapy, since it has been reported to be expressed in most cases of AML, both on bulk leukemic cells and LSC subpopulation, with higher expression potentially associated with poorer prognosis [1,65,87]. Perriello et al., recently documented that CD123 was highly expressed on *NPM1*-mutated AML cells both at diagnosis and relapse, with the highest levels detectable in cases carrying concomitant *FLT3*-ITD [87]. High CD123 expression levels were consistently found on *NPM1*-mutated CD34+ CD38- putative LSC, suggesting that immunotherapies targeting CD123 could potentially be particularly effective in *NPM1*-mutated AML patients [1,65,87]. However, due to the low expression of CD33 or CD123 on healthy tissues, including normal hematopoietic stem cells, targeting these surface antigens through either monoclonal antibodies/T-cell recruiting antibody constructs or CAR-T cells may be hampered by potentially threatening “on-target/off-tumor” toxicity, including myeloablation [1,65,87].

## 8. Conclusions

**Figure 1:**
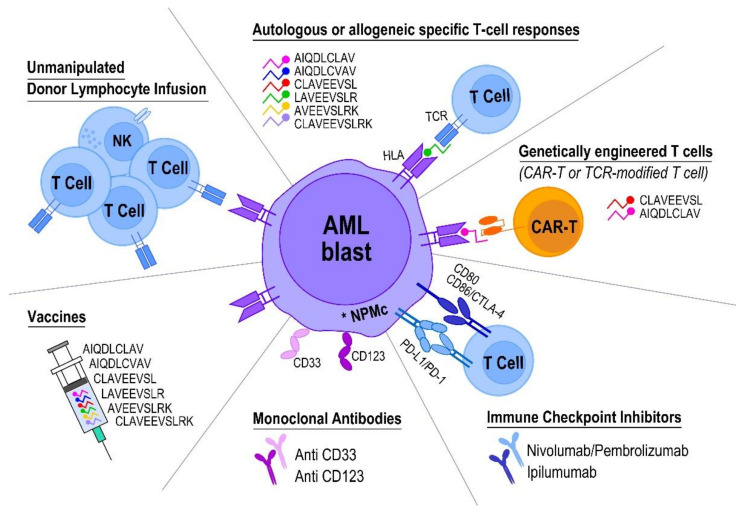
Exploiting immunotherapy against NPM1-mutated AML.Autologous or allogeneic NPM1-mutated-specific T-cell responses can naturally occur in patients receiving either conventional chemotherapy/hypomethylating agents or allogeneic HSCT, respectively. Adoptive immunotherapy carried out by infusion of NPM1-mutated-specific T cells, stimulated and expanded directly from patients, could potentially be promising in maintaining complete remission or eradicating persistent measurable residual disease in subjects not candidate to allogeneic HSCT. The selected NPM1-mutated-derived peptides that are most immunogenic are listed. In addition to unmanipulated DLI, neoantigen-specific DLI obtained from healthy donors could represent a therapeutic option in case of morphological or molecular AML relapse after allogeneic HSCT. Genetically engineered T cells directed against NPM1-mutated-derived peptides bound to HLA may also represent a newer immunotherapeutic strategy. Immune-checkpoint inhibitors and NPM1-mutated peptide vaccines could also prospectively be considered to further elicit and stimulate NPM1-mutated-specific immune responses. Finally, CD33 and CD123, antigens strongly expressed on NPM1-mutated blast cell surface, are currently recognized as valuable targets for monoclonal antibodies/T-cell recruiting antibody constructs or CAR-T cells, despite being hampered by “on-target/off-tumor” toxicity.

*NPM1* mutations are recurrent molecular lesions, occurring in nearly 30% of AML patients, are stable across the disease course and are considered to be driver events, highly specific for leukemogenesis [5,7,8,9,10]. Indeed, NPM1-mutated C-terminal aminoacidic sequences are not found in either malignancies different from AML or normal tissues, resulting in leukemia-specific neoantigens considered optimal target for immunotherapy [2,3,15,16,19,20,88,89]. Despite the globally immunosuppressive BM microenvironment, the spontaneous development of endogenous specific anti-leukemic T-cell immunity directed against highly immunogenic NPM1-mutated-derived peptides has been observed in several patients with *NPM1*-mutated AML and may contribute to the maintenance of long-lasting CR and prolonged survival [16,19,27]. Whether the frequency and intensity of NPM1-mutated-specific T-cell responses may vary according to the molecular landscape found in *NPM1*-mutated AML patients still needs to be elucidated. In a small patient subgroup, Forghieri et al., did not document significantly different amounts of specific immune response against NPM1-mutated peptides, when cases were compared according to *FLT3* mutational status [19]. However, the impact of additional molecular lesions on NPM1-mutated-specific T-cell responses warrants further investigations in larger patient series. Relevant to this, Mer et al., recently identified two distinct subtypes within *NPM1*-mutated AML patients, referred to as “primitive” or “committed”, based on the respective presence or absence of a stem cell signature [90]. Using gene expression profiling, epigenomic and immunophenotyping, each subtype was associated with particular molecular characteristics, disease differentiation state, and patients survival. Of note, in the “committed” subtype, immunomodulatory genes, such as CD163 and CD14, were up-regulated. Additionally, immune response pathways such as IFNγ-mediated signaling, GPCR signaling, and toll-like receptor signaling were up-regulated in this disease subgroup, with potential implications also in occurrence or persistence of NPM1-mutated-specific immune responses, to be prospectively investigated [90]. Although generally associated with favorable prognosis, especially in the absence of *FLT3*-ITD, approximately 50% of *NPM1*-mutated AML patients receiving conventional treatment approaches, based on chemotherapy and HSCT procedures, still currently die due to disease relapse and progression [7,8,87]. Careful monitoring of the correlation between NPM1-mutated transcripts MRD and specific T-cell responses against NPM1-mutated peptides, easily evaluable in PB samples, may provide relevant prognostic information [19]. Beyond novel agents either targeting abnormal cell transport of NPM1-mutated protein, such as XPO1 inhibitors, or targeting HOX expression, namely the menin-MLL inhibitors MI3454 and VTP-50469, or triggering nucleolar stress, such as dactinomycin, which have shown anti-leukemic activity in pre-clinical models and have started to be investigated in humans, immunotherapeutic approaches targeting NPM1-mutated protein processed and presented by HLA system on AML cell surface could represent an effective treatment option, at least in some distinct disease phases [7,91,92]. Due to diverse bioinformatic instruments and in vitro/ex vivo immunological platforms employed, investigators identified different epitopes from NPM1-mutated protein showing the potentially highest immunogenicity, as detailed in Table 1 [4,15,16,18,19,20,21,25]. In order to expand the opportunity to track the occurrence and clinical significance of NPM1-mutated-specific T-cell immunity, it could be possibly suggested to utilize for future immunological experiments a combination of at least AIQDLCLAV, AIQDLCVAV, CLAVEEVSL, LAVEEVSLR, AVEEVSLRK 9-mer, and CLAVEEVSLRK 11-mer immunogenic peptides, representative of the more common *NPM1* mutation types and able to efficiently bind to at least most common HLA types, such as A*02:01 and A*03:01, which are frequently found in the Caucasian population (Table 1) [4,15,16,18,19,20,21,25]. The use of engineered T cells, namely CAR-T or TCR-transduced T cells, directed against NPM1-mutated peptides bound to HLA could represent a promising therapeutic approach, warranting future investigation on the potential clinical application [3,4,7,19,51]. Although either adoptive or vaccine-based immunotherapies to elicit endogenous immune responses are unlikely to be highly effective in patients with full-blown either newly diagnosed or relapsed leukemia, these strategies, potentially in combination with ICPIs, could be promising in maintaining CR or preemptively eradicating persistent MRD, following conventional chemotherapy in older *NPM1*-mutated AML patients not eligible for allogeneic HSCT [19,21,42,93]. Alternatively, neoantigen-specific DLI derived from healthy donors and targeting NPM1-mutated protein to selectively elicit GvL may be an attractive therapeutic option in subjects experiencing morphological or, preferentially, molecular relapse after allogeneic HSCT, as previously demonstrated in Philadelphia chromosome-positive B-acute lymphoblastic leukemia patients (Figure 1) [19,37,58,59,61,62,94,95]. In conclusion, even though it has thoroughly been documented that NPM1-mutated protein is immunogenic and may elicit neoantigen-specific immune responses in vivo, further prospective studies are warranted to investigate whether individualized anti-leukemic immunotherapeutic approaches could have a potential clinical utility in *NPM1*-mutated AML patients throughout their disease course [1,3,4,19,20,70].

## Figures and Tables

**Table 1:** Selection of most immunogenic NPM1-mutated epitopes.

Reference	Amino Acid Sequences	Position	*NPM1* Mutation Types	HLA Binding Restriction	Peptide Identification
Liso et al., 2008 [15]	CLAVEEVSL (9-mer) AIQDLCLAV (9-mer)	288–296 283–291	A/D/G/H A/D/G/H	A*02:01 A*02:01	in silico and in vitro
Greiner et al., 2012 [16]	AIQDLCLAV (9-mer) AIQDLCVAV (9-mer)	283–291 283–291	A/D/G/H C	A*02:01 A*02:01	in silico and ex vivo
Kuzelova et al., 2015 [18]	LAVEEVSL (8-mer) QEAIQDLCLAV (11-mer) AVEEVSLRK (9-mer) LAVEEVSLR (9-mer) QEAIQDLCL (9-mer)	289–296 281–291 290–298 289–297 281–289	A/D/G/H A/D/G/H A/B/C/D/G/H/J A/D/G/H A/D/G/H	C*03:03/C*03:04/B*35:03 B*37:01/B*49:01/B*40:02 A*11:01 A*68:01 B*40:01	in silico
Ruecker-Braun et al., 2016 [24]	AIQDLCLAV (9-mer)	283–291	A/D/G/H	A*02:01	ex vivo
Kuzelova et al., 2018 [25]	CLAVEEVSL (9-mer) DLCLAVEEV (9-mer) AIQDLCLAV (9-mer)	288–296 286–294 283–291	A/D/G/H A/D/G/H A/D/G/H	A*02:01 A*02:01 A*02:01	in silico
Forghieri et al., 2019 [19]	LAVEEVSLR (9-mer) AVEEVSLRK (9-mer) CLAVEEVSLRK (11-mer)	289–297 290–298 288–298	A/D/G/H A/B/C/D/G/H/J A/D/G/H	AVEEVSLRK known to have in silico binding affinity for HLA-A*02:01/A*03:01/ A*11:01/A*68:01	ex vivo
van der Lee et al., 2019 [20]	CLAVEEVSL (9-mer) VEEVSLRK (8-mer) AVEEVSLR (8-mer) AVEEVSLRK (9-mer) CLAVEEVSLRK (11-mer)	288–296 291–298 290–297 290–298 288–298	A/D/G/H A/B/C/D/G/H/J A/B/C/D/G/H/J A/B/C/D/G/H/J A/D/G/H/J	A*02:01 - - A*03:01/A*11:01 A*03:01/A*11:01	in vitro, ex vivo and in vivo (murine model)
Narayan et al., 2019 [21]	AVEEVSLRK (9-mer) C(Cys)LAVEEVSL (9-mer)	290–298 288–296	A/B/C/D/G/H/J A/D/G/H	A*03:01/A*11:01/A*31:01/ A*66:01/A*68:01/A*30:01 A*02:01	in silico and in vitro
Xie et al., 2020 [4]	AIQDLCLAV (9-mer)	283–291	A/D/G/H	A*02:01	in vitro and in vivo (mouse model)

NPM1, Nucleophosmin; HLA, Human Leukocyte Antigen.

## Data Availability

Not applicable.

## Abbreviations

AML	acute myeloid leukemia
HSCT	hematopoietic stem cell transplantation
DLI	donor lymphocyte infusion
NPMc	nucleophosmin1 with abnormal cytoplasmic delocalization
CAR-T	chimeric antigen receptor-T
TCR	T-cell receptor
HLA	human leukocyte antigen
